# Fetal haemoglobin and oxygen requirement in preterm infants: an observational study

**DOI:** 10.1136/archdischild-2024-327411

**Published:** 2024-09-25

**Authors:** Tommy Ulinder, William Hellström, Christian Gadsbøll, Linda Nilsson, Margareta Gebka, Gustav Robertz, Matteo Bruschettini, Ann Hellstrom, David Ley

**Affiliations:** 1 Department of Clinical Sciences Lund, Paediatrics, Lund University, Lund, Sweden; 2 Skåne University Hospital, Lund, Sweden; 3 Department of Pediatrics, Institute of Clinical Sciences, Sahlgrenska Academy, University of Gothenburg, Gothenburg, Sweden; 4 Department of Clinical Sciences Lund, Paediatrics, Lund University, Skane University Hospital, Lund, Sweden; 5 Department of Paediatrics, Ystad Hospital, Ystad, Sweden; 6 Ophthalmology, Sahlgrenska Academy, Goteborg, Sweden

**Keywords:** Neonatology, Paediatrics, Intensive Care Units, Neonatal

## Abstract

**Objective:**

To investigate the relationship between the fraction of fetal haemoglobin (HbF(%)) and oxygen requirement as determined by the fraction of inspired oxygen (FiO_2_) and alveolar–arterial gradient (A–a gradient). Increased alveolar exposure to oxygen may explain the association between decreased HbF(%) and the development of bronchopulmonary dysplasia (BPD).

**Design:**

Longitudinal, retrospective, observational study.

**Setting:**

Tertiary-level neonatal intensive care unit, referral centre for southern Sweden.

**Patients:**

Four hundred forty very preterm infants born before gestational week 30, 2009–2015.

**Intervention:**

Regular clinical practice.

**Main outcome measures:**

The FiO_2_ and A–a gradient were determined at the time-point of 10 015 arterial blood gas analyses obtained during postnatal days 1–7. The relationship between HbF(%) and FiO_2_ and A–a gradient and the modifying influence of other factors affecting haemoglobin oxygen affinity were evaluated.

**Results:**

We found a significant relationship between a low fraction of HbF and an increase in FiO_2_ and A–a gradient, respectively. These relationships remained significant after adjusting for pH, pCO_2_, postnatal age, gestational age and sex.

**Conclusion:**

These high-resolution data show that decreased HbF(%) during the first postnatal week is associated with increased FiO_2_ and A–a gradient in very preterm infants. Increased alveolar exposure to oxygen and resulting oxidative stress may, at least partly, explain the previously reported associations between decreased HbF, blood transfusions and the development of BPD in preterm infants.

**WHAT IS ALREADY KNOWN ON THIS TOPIC?**

- There is a well-described association between low fraction of fetal haemoglobin during the first weeks of life and neonatal morbidity such as retinopathy of prematurity and bronchopulmonary dysplasia (BPD).
- In very preterm infants, HbF is rapidly replaced by adult haemoglobin due to frequent adult donor blood transfusions.

**WHAT THIS STUDY ADDS?**

- We show that low HbF(%) is associated with increased oxygen requirement, exposing the immature alveolar tissue to an augmented hyperoxic environment inducing oxidative stress and inflammation.
- These findings might partly provide a mechanistic link between low HbF(%) and BPD.

**HOW THIS STUDY MIGHT AFFECT RESEARCH, PRACTICE OR POLICY**

- Prospective randomised intervention trials are urgently needed to study the effects of minimising iatrogenic blood loss and maintaining circulating levels of HbF in extremely preterm infants.

## Introduction

 Fetal blood contains factors that are essential for normal development.^1^ Such factors include fetal haemoglobin (HbF). Low circulating levels of HbF during early postnatal development have been associated with neonatal morbidity.^2^,^5^ In preterm infants, levels of HbF decrease gradually after birth, and in infants born at term, HbF is less than 10% of total Hb by the age of 16 weeks.^6^ Other data suggest a natural decline of HbF with a weekly rate of 16%, reaching a complete switch from HbF to adult Hb (HbA) after 25 weeks following birth.^7^

Preterm infants are often subject to neonatal intensive care, which involves frequent blood sampling. In a study investigating blood loss in extremely preterm infants, blood sampling during the first 2 weeks of life corresponded to an iatrogenic blood loss of, on average, 40 mL/kg corresponding to 58% of the total blood volume.^8^

As a result of iatrogenic blood loss, infants are exposed to frequent red blood cell transfusions. Adult donor blood thus replaces fetal blood, and HbF(%) rapidly decreases with each transfusion. Several reports have described associations between red blood cell transfusion and neonatal morbidities, including necrotising enterocolitis (NEC), retinopathy of prematurity (ROP) and bronchopulmonary dysplasia (BPD).^9^,^11^ It has previously been shown that an early postnatal decrease in HbF(%) is associated with the development of BPD in a cohort of very preterm infants. The probability of BPD was inversely related to mean HbF(%) during the first postnatal week, with a probability of 20% for the development of BPD at a mean HbF(%) of 80% and a 90% probability at a mean HbF(%) of 40%.^3^ The potential causal mechanisms involved in BPD development reflected by changes in HbF would thus appear to be operative during early postnatal development.

Several physiological factors including pCO_2_, pH and type of Hb modify the affinity of oxygen to Hb. HbA has a lower affinity to oxygen than HbF, shifting the oxyhaemoglobin dissociation curve to the right. The replacement of HbF by HbA due to blood transfusions from adult donors and the anticipated higher systemic tissue exposure to dissolved oxygen have been proposed as a plausible cause of ROP. Conversely, a corresponding shift of HbF towards predominantly HbA in the pulmonary circulation will lead to a requirement of higher intra-alveolar oxygen concentrations to maintain blood-borne oxygen saturation. The relationship between intra-alveolar and arterial oxygen tension is sensitively measured by the alveolar–arterial (A–a) gradient, which is defined as the difference between the alveolar and the arteriolar concentrations of oxygen and is a highly accurate index of pulmonary function.

We hypothesised that a shift from predominantly HbF to HbA, with its lower oxygen affinity, due to transfusions with adult donor blood, is associated with an increased oxygen requirement to maintain peripheral oxygen saturations within the desired target range (91–95%). We further hypothesised that the association between the shift of type of Hb and oxygen requirement remains independent of other physiological factors affecting haemoglobin oxygen affinity. This was investigated by evaluating the relationship between fraction of inspired oxygen (FiO_2_), A–a gradient and HbF(%) in 440 inborn very preterm infants during the first week of life.

## Materials and methods

### Patients and clinical characteristics

We conducted a single-centre retrospective observational cohort study including 440 infants born before 30 gestational weeks at Skåne University Hospital in Lund and admitted to tertiary-level neonatal intensive care between 2009 and 2015. There were no exclusion criteria. The outcome was oxygen requirement as determined by FiO_2_ and A–a gradient with HbF(%) being the exposure variable. Modifying variables were gestational age (GA), postnatal age (PNA), PaCO_2_, sex and pH.

GA was determined by ultrasound at postmenstrual week 17–18. Birth weight (BW) was obtained from medical birth charts and small for gestational age was defined as BW more than 2 SD below the GA-related mean of the population according to the Swedish reference curve for normal growth.^12^

Infants received supplemental oxygen aiming to maintain a target level of peripheral arterial saturation (SpO_2_) between 91% and 95%. Red blood cell transfusions were administered to keep total Hb above 140 g/L during the first week of life.

BPD was defined by the requirement of supplementary oxygen at 36 postmenstrual weeks.

### Blood gas analyses

HbF(%), haemoglobin (Hb g/L), pH, oxygen saturation (SaO_2_ %), and partial oxygen (PaO_2_ kPa) and carbon dioxide (PaCO_2_ kPa) tensions were obtained from arterial blood gas analyses performed during PNA days 1–7. Venous and capillary blood gas analyses were excluded from the study. Data was retrieved from the local hard drive of the blood gas analyser (Radiometer 800, Copenhagen, Denmark). FiO_2_ at the time-point of each arterial blood gas analysis was registered. Chart data on FiO_2_ at the time-point of blood gas withdrawal were available for 9014 blood gas analyses.

Alveolar oxygen partial pressure (PAO_2_) was calculated using the alveolar gas equation.

PAO_2_ = (Patm – PH_2_O) FiO_2_ – PaCO_2_/RQ

RQ=respiratory quotient. RQ is the amount of CO_2_ produced/the amount of O_2_ consumed.^13^

### Statistics

Random coefficient models, to avoid ascertainment bias, were applied for longitudinal description of HbF(%), FiO_2_ and A–a gradient, as a function of PNA, including sex and GA categories, and significant interactions. HbF(%) was modelled using a normal distribution, and the other variables were modelled using a lognormal distribution due to skewness in the data. The number of infants in the respective GA categories were 22–24 weeks (n=116), 25–26 weeks (n=230) and 28–29 weeks (n=94). An unstructured covariance pattern was used, and time, that is*,* PNA, was modelled using natural cubic splines. Additionally, FiO_2_, A–a gradient and SaO_2_ were expressed as a function of HbF(%), including pCO_2_, GA and PNA, adjusted for sex and pH using the same methodology. A decrease in outcome variables over increasing HbF(%) was described by relative risk and 95% CI and associated p-values for FiO_2_ and A–a gradient and by mean difference and 95% CI and associated p values for SaO_2_. Diagnostic plots were visually reviewed for goodness-of-fit and appropriateness of the methods used. All interactions (HbF(%), sex, GA, PNA, pCO_2_ and pH) were investigated, and those that were significant were kept in the model.

## Results

Four hundred forty infants were included in this observational study. The mean (SD) GA was 25.9 (SD 1.8) weeks, with a mean (SD) BW of 876 (274) g. Mortality reported as death before 36 weeks of postmenstrual age was 11.8% (n=52). Three hundred seventy-nine infants survived until postmenstrual age 36 weeks and were thus eligible for BPD diagnosis. Two hundred eleven infants (55.7 %) developed BPD. Clinical characteristics are presented in table 1.

**Table 1:** Clinical characteristics of infants (n=440) live-born before 30 weeks of gestation, delivered at Skåne University Hospital in Lund and admitted to a tertiary-level neonatal intensive care unit (NICU) in Lund between 2009 and 2015

GA weeks; mean (SD)	25.9 (1.8)
GA 22–24 weeks; n (% of total cohort)	116 (26.4)
GA 25–27 weeks; n (% of total cohort)	230 (52.3)
GA 28–29 weeks; n (% of total cohort)	94 (21.4)
Birth weight, g; mean (SD)	876 (274)
SGA; n (%)	105 (23.9)
Mortality*	52 (11.8)
Males; n (%)	250 (56.8)
Twins; n (%)	106 (24.1)
Triplets; n (%)	18 (4.1)
Morbidities:	
NEC; n (%)†	20 (4.8)
BPD; n (%)‡	211 (55.7)
Any IVH; n (%)§	119 (27.8)
IVH 3–4; n (%)§	42 (9.8)
Any ROP; n (%)¶	103 (23.4)

* Before 36 weeks postmenstrual age.

† 420 available for analysis., 379 available for analysis, 428 available for analysis, 382 available for analysis.

‡ 379 available for analysis.

§ 428 available for analysis.

¶ 382 available for analysis.

.BPD, bronchopulmonary dysplasia; GA, gestational age; IVH, intraventricular haemorrhage; n, number; NEC, necrotising enterocolitis; ROP, retinopathy of prematurity; SGA, small for gestational age.

The total number of blood gas analyses was 10 015 distributed per postnatal day (PND); PNA day 1 (n=2152), PNA day 2 (n=1916), PNA day 3 (n=1586), PNA day 4 (n=1345), PNA day 5 (n=1228), PNA day 6 (n=1170) and PNA day 7 (n=618).

**Figure 1:**
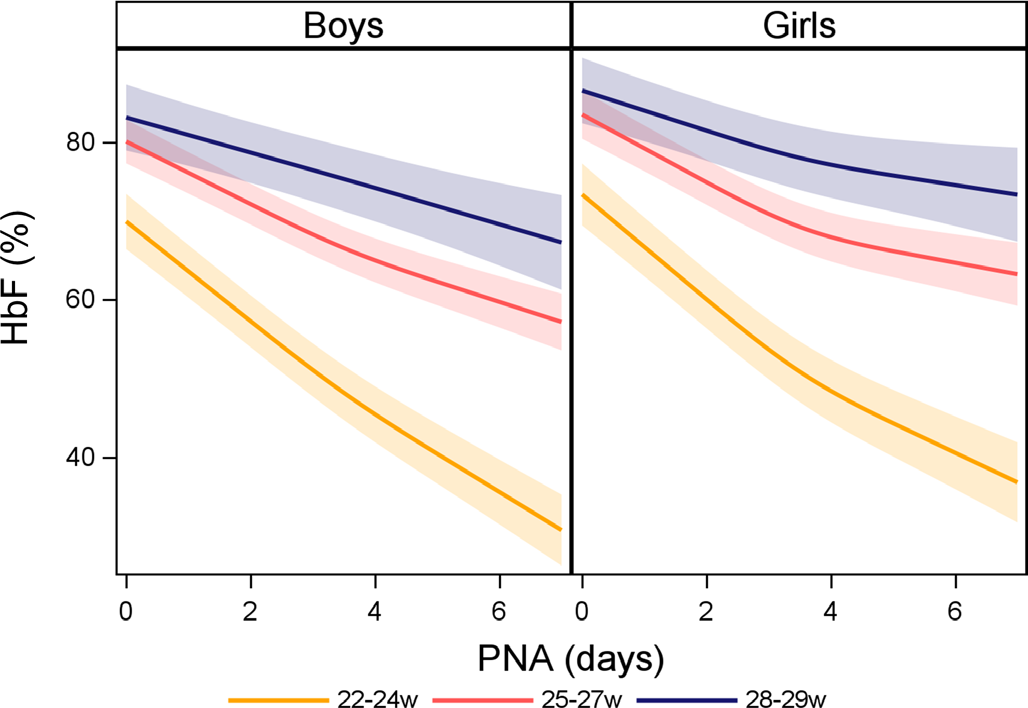
Fetal haemoglobin (HbF) (%) over postnatal age (PNA) by gestational age category and sex. The shadowed areas illustrate a 95% CI. In total, 10 015 arterial blood gases were analysed in 440 infants born consecutively before gestational week 30 (2009–2015). w, weeks.

Evaluating HbF(%) in relation to PNA, GA at birth and sex showed a decline of HbF(%) in relation to increasing PNA, which was increasingly prominent with decreasing GA. The decline of HbF(%) in relation to PNA was similar in girls and boys (figure 1, online supplemental table 1).

**Figure 2:**
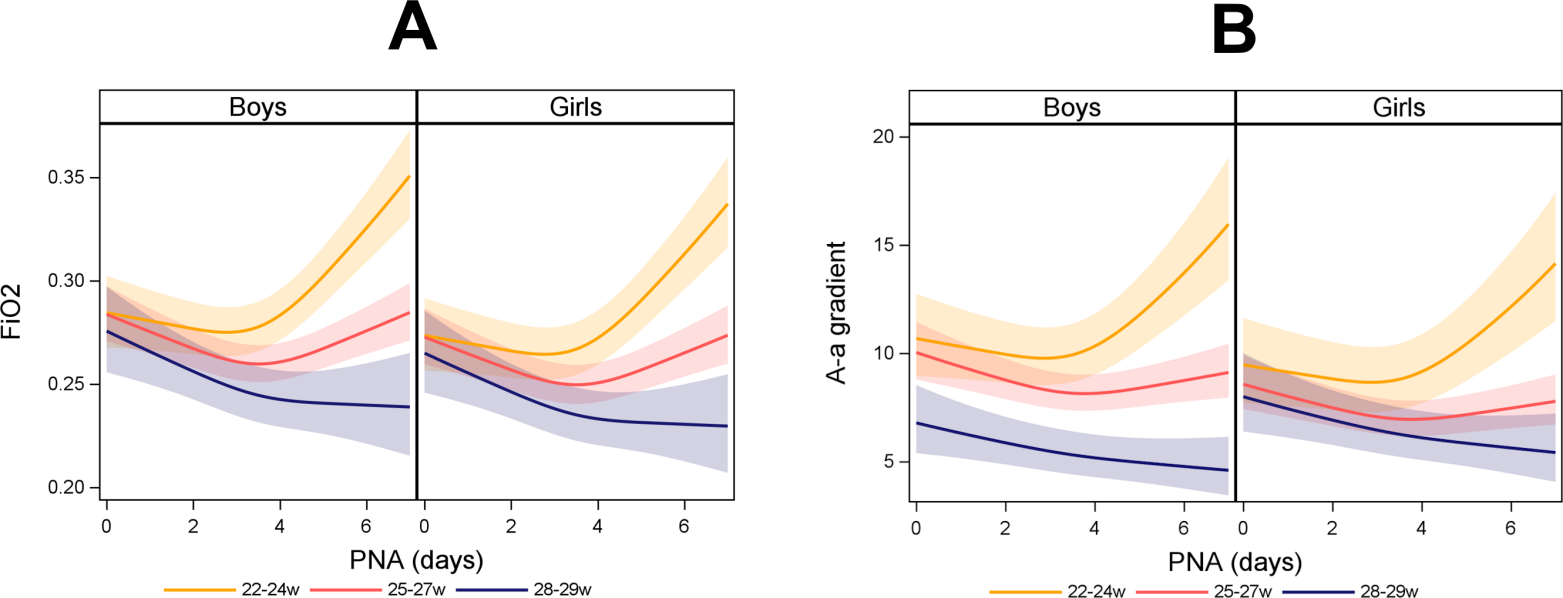
(**A and B**). FiO_2_ and alveolar–arterial (A–a) gradient over postnatal age (PNA) by gestational age (GA) at birth category and sex. Time modelled with natural cubic splines because the effect of GA and sex varied over time. Shadowed areas illustrate 95% CI. In total, 10 015 arterial blood gases were analysed in 440 infants born consecutively before 30 gestational week (2009–2015). GA 22–24 weeks (n=116), GA 25–27 weeks (n=230) and GA 28–29 weeks (n=94). w, weeks.

Oxygen requirement, as determined by FiO_2_ and A–a gradient, respectively, decreased from birth until PNA 3 days in all GA subgroups with a clear shift to an increase from PNA 3 days to PNA 7 days in subgroups with low GA as opposed to a continued decrease in the most mature subgroup of infants (figure 2, online supplemental table 2.1, 2.2).

**Figure 3:**
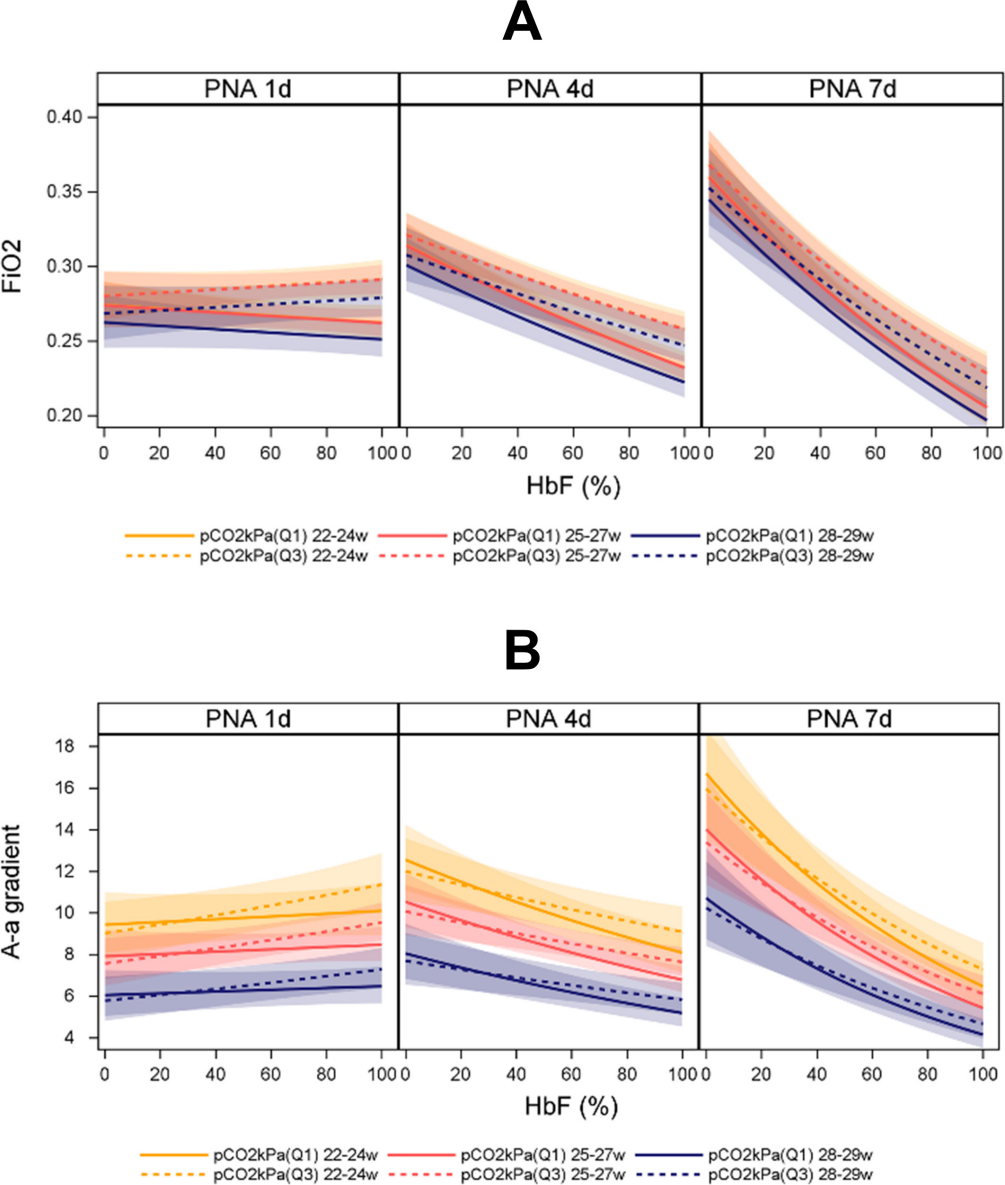
(**A and B**). FiO_2_ and alveolar–arterial (A–a) gradient as a function of HbF (%), pCO_2_, gestational (GA) and postnatal age (PNA) adjusted for pH and sex. Shadowed areas illustrate 95% CI. In total, 10 015 arterial blood gases were analysed in 440 infants born consecutively before 30 gestational week (2009–2015). Q1 and Q3 of pCO_2_ represent the 25th and 75th percentile, respectively. GA 22–24 weeks (n=116), GA 25–27 weeks (n=230), GA 28–29 weeks (n=94). PNA day 1 (n=2152), PNA day 4 (n=1345), PNA day 7 (n=618). HbF, fetal haemoglobin; n, number; Q, quartile; w, weeks.

We then evaluated the influence of HbF(%) on the respective determinants of oxygen requirement in relation to PNA, GA at birth and pCO_2_, adjusted for pH and sex. FiO_2_ and A–a gradient, respectively, were inversely associated with HbF(%), and these relationships showed a strong interaction with PNA. The inverse association became more apparent with increasing PNA with an approximately 10% and 16% reduction of FiO_2_ and A–a gradient, respectively, associated with an average 20% increase in HbF(%) on PND 7 (figure 3, online supplemental table 3.1, 3.2).

**Figure 4:**
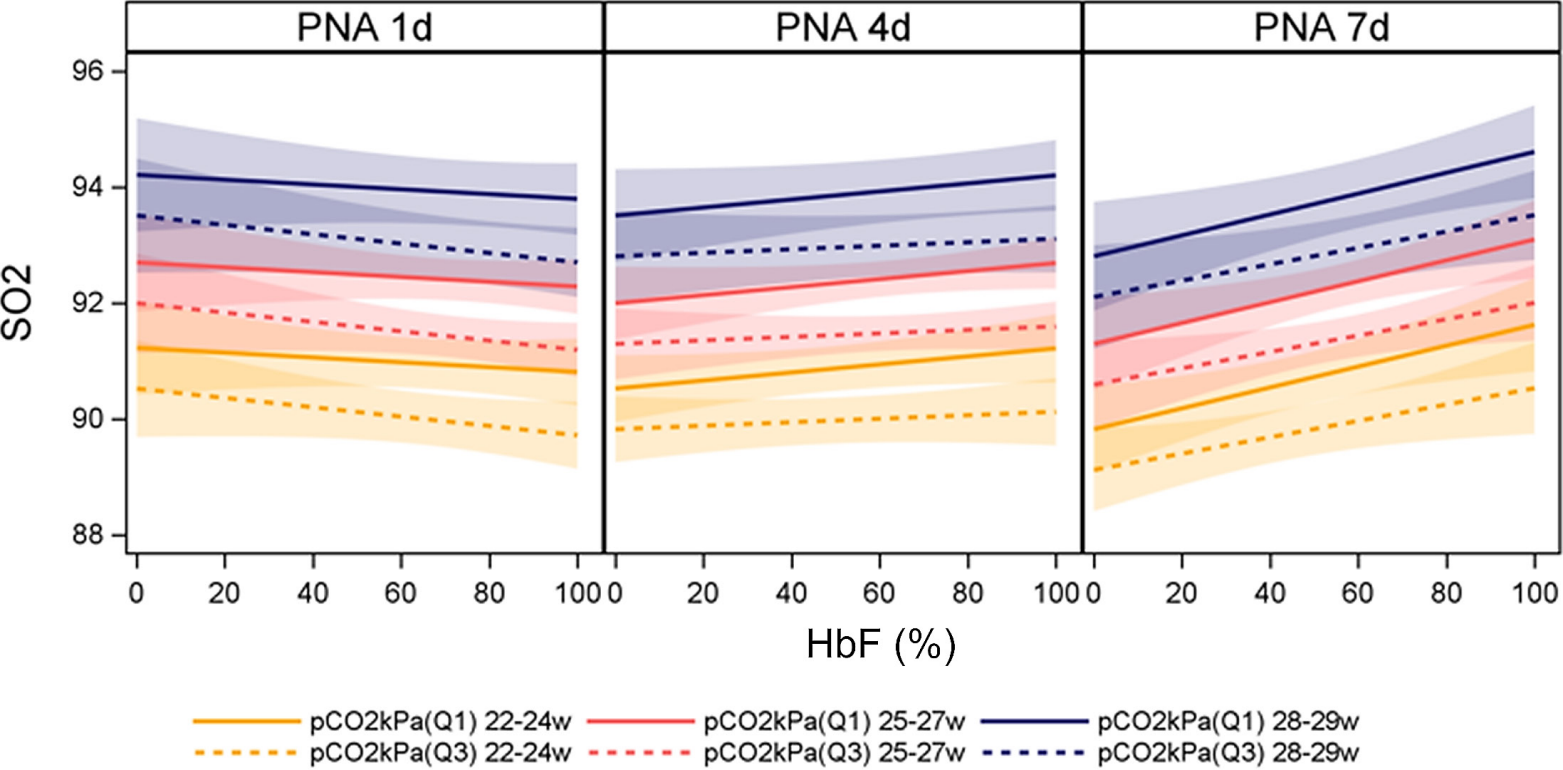
Arterial oxygen saturation (SaO_2_) as a function of HbF (%), pCO_2_, gestational age (GA) and postnatal age (PNA) adjusted for pH and sex. Q1 and Q3 of pCO_2_ represent the 25th and 75th percentile, respectively. GA 22–24 weeks (n=116), GA 25–27 weeks (n=230), GA 28–29 weeks (n=94). In total, 10 015 arterial blood gases were analysed in 440 infants born consecutively before 30 gestational week (2009–2015) . PNA day 1 (n=2152), PNA day 4 (n=1345), PNA day 7 (n=618). HbF, fetal haemoglobin; q, quartile; w, weeks.

Concentrations of administered inhaled oxygen were based on monitored peripheral SO_2_ (SpO_2_) obtained by pulse oximetry with a desired range between 91% and 95%. Biased measurements by pulse oximetry towards higher SpO_2_ values in infants with higher HbF(%) may have confounded the evaluated associations between HbF(%) and indices of oxygen requirement.^14^,^16^ We therefore evaluated the association between HbF(%) and SaO_2_ in relation to PNA, GA at birth and pCO_2_, adjusted for pH and sex. There was no significant adjusted relationship between HbF(%) and SaO_2_ at PND 1 and PND 4 with a moderately positive association at PND 7 corresponding to an average increase of 0.32% in mean aSO_2_ with a 20% increase in HbF (figure 4, online supplemental table 4). Thus, the observed relationship between reduced oxygen requirement in association with higher HbF(%) was not confounded by biased pulse oximetry measurements.

## Discussion

This study aimed to provide a link for the previously observed association between decreased levels of HbF during the first postnatal week and the development of BPD.^3^ In this cohort of 440 infants born before gestational week 30, we observed that reduced circulating HbF(%) was associated with increased inhaled oxygen requirement as determined by FiO_2_ and calculated A–a gradient during the first postnatal week. These data suggest that the decreased oxygen affinity of HbA may play a causal role by amplifying alveolar exposure to inhaled oxygen in preterm infants. Notably, the relationship between HbF(%) and alveolar oxygen exposure was independent of other factors influencing Hb oxygen affinity, namely pCO_2_ and pH.

The general assumption has been that an exchange of predominantly HbF by HbA due to red blood cell transfusion leads to an increased exposure of systemic tissues to oxygen, that is, increased levels of oxygen dissolved in plasma measured by PaO_2_, explained by the lower affinity of HbA for oxygen.^17^ In line with this, circulating biomarkers of oxidative stress were significantly higher in preterm infants with lower HbF levels.^18^ However, our previous data in the present cohort showed that the development of BPD was associated with decreased arterial PaO_2_,^3^ which contradicts the general assumption. We, therefore, hypothesised that the decreased affinity of HbA to oxygen in the pulmonary circulation plays a primary role and potentially overwhelms systemic aspects.

Our findings indicate that decreased HbF(%) is associated with a higher exposure of alveolar tissue to oxygen. The fetal lung is programmed to mature in a hypoxic environment in utero. During postnatal transition, this environment changes drastically towards becoming relatively hyperoxic, even on room air with 21% oxygen. It is generally accepted that oxygen-induced lung injury is mediated by reactive oxygen species (ROS) generated by normal oxidative metabolism within the cell. The generation of ROS is exacerbated in the presence of inflammation and hyperoxia. There is a delicate balance between the generation of ROS and the presence of antioxidants to defend the cell in vivo. This balance is disturbed in the presence of hyperoxia, inflammation and reperfusion of ischaemic tissue.^19^ Preterm lung tissue has limited antioxidant ability and is exposed to hyperoxia and inflammation. We now know that infants with low HbF(%) constitute a risk group for augmented alveolar hyperoxia and are thus at risk of further ROS production and antioxidant ability imbalance.

It is clinically known that after initial stabilisation, supplemental oxygen requirement in the most premature infants usually remains at low levels for the first couple of days and then increases. This was clearly demonstrated in the present study (figure 2). Notably, the postnatal age for an increase in the requirement of inhaled supplemental oxygen in the most immature infants coincided with the inverse association between HbF(%) and oxygen requirement becoming apparent.

Studies in animal models have demonstrated that exposure of lung tissue to high levels of oxygen leads to endothelial and epithelial destruction followed by pulmonary oedema, haemorrhage, alveolar epithelial type 2 cell hyperplasia and fibrosis, all of which compromise gas exchange over the alveolar membrane.^20^ The A–a gradient is a good indicator for determining the underlying cause of hypoxia. If hypoxia is caused by hypoventilation, the A–a gradient will remain normal since both PAO_2_ and PaO_2_ will decrease. If the reason for hypoxia is intrapulmonary, the A–a gradient will increase since PAO_2_ will increase because of compromised gas exchange.^21^

The result of this observational study, a significant increase in the A–a gradient with falling HbF(%), may be interpreted as evidence of early alveolar damage due to increased oxygen exposure. The relationship between HbF(%) and oxygen requirement, as determined by FiO_2_ and the A–a gradient, became more evident over the first week of life, being most significant on PND 7. This change may represent a continuous inflammatory and oxidative process in the alveolar compartment of infants subjected to repeated systemic replacement of HbF with HbA.

Jiramongkolchai *et al*^22^ reported an association between low levels of HbF, ROP and poor oxygenation indices in a small prospective cohort of preterm infants. They further observed a correlation between low HbF(%) and high oxygen requirement at isolated time points. The present study showed that the relationship between decreased HbF(%) and augmented alveolar oxygen exposure was evident already during the first postnatal week. Previous studies have indicated that pulse oximetry monitoring of oxygen saturation is prone to measurement error at varied HbF(%) with a bias towards overestimating oxygen saturation at higher HbF(%). Thus, false high pulse oximetry readings may have resulted in insufficient administered inhaled oxygen concentrations in infants with high HbF(%). However, this was not the case as SaO_2_ did not decrease in relation to HbF(%) during the first postnatal week.

The main limitation of the study is the retrospective, single-centre design. Data was collected at one tertiary centre in Sweden over 7 years, and clinical procedures may have been modified during the period. All consecutive inborn infants delivered before gestational week 30 were included. The retrospective character of the study increases the risk of confounding by indication, with the sickest infants being subjected to the most frequent blood sampling and subsequent blood transfusions. However, we took great care to adjust for relevant confounding factors, but the possibility of residual confounding cannot be ruled out. Adjustment for intraerythrocytic 2,3-diphosphoglycerate was impossible. We had no information about mean airway pressure and mechanical ventilation and could thus not adjust for this even though this might influence FiO_2_. The study’s main strengths are the size of the study cohort and the large number of blood gas analyses obtained. The statistical analysis strategy employed aimed to maximise statistical power and avoid stratification with resulting subgroup analysis. All blood gas analyses were performed and downloaded from the same blood gas analyser throughout the study, minimising the risk of inter-equipment differences and data transfer errors.

## Conclusion

Decreased HbF(%) was associated with increased inhaled oxygen requirement during the first postnatal week in very preterm infants. This association remained strong after adjustment for other factors with a modifying influence on Hb oxygen affinity. Increased oxygen exposure in the alveolar space may aggravate oxidative stress and inflammation and thus be causal in BPD development. Measures directed at counteracting premature replacement of HbF with HbA may prevent neonatal morbidity. An ongoing, multicentre, prospective randomised controlled trial (https://clinicaltrials.gov/study/NCT04239690?) is currently evaluating the effects of minimising iatrogenic blood loss in extremely preterm infants on neonatal morbidity.

## Supplementary material

10.1136/archdischild-2024-327411 online supplemental file 1

## Data Availability

Data are available upon reasonable request.
