# Supplementary material for: Fetal haemoglobin and oxygen requirement in preterm infants: an observational study
Source: Arch Dis Child Fetal Neonatal Ed. 2024 Sep 25;110(3):e327411. doi: 10.1136/archdischild-2024-327411 (PMC12013580; doi:10.1136/archdischild-2024-327411)
Supplement: online supplemental file 1 [file fetalneonatal-110-3-s001.pdf]

SUPPLEMENTARY TABLES

Suppl. Table 1. Statistical model for HbF % over postnatal age (PNA) using random coefficient models, with normal distribution and unstructured covariance pattern, including significant interactions. Time modeled with natural cubic splines.

Variable	F value	p-value	PNA spline	GA cat.	Estimate	SE	95% LCL	95% UCL	t-value	p-value
PNA spline	37.66	<.0001	1		0.000					
			2		-1.900	0.681	-3.237	-0.564	-2.792	0.0054
			3		-0.286	0.160	-0.600	0.028	-1.785	0.07
GA cat.	16.66	<.0001		22-24w	-13.193	2.562	-18.227	-8.160	-5.150	<.0001
				25-27w	-3.045	2.278	-7.521	1.431	-1.337	0.18
				28-29w	0.000					
Sex	3.78	0.052			3.424	1.761	-0.037	6.885	1.944	0.052
PNA spline * GA cat.	18.72	<.0001	1	22-24w	0.000					
			1	25-27w	0.000					
			1	28-29w	0.000					
			2	22-24w	-4.105	0.535	-5.155	-3.055	-7.674	<.0001
			2	25-27w	-1.751	0.493	-2.718	-0.783	-3.550	0.0004
			2	28-29w	0.000					
			3	22-24w	0.298	0.130	0.043	0.554	2.287	0.022
			3	25-27w	0.287	0.128	0.035	0.538	2.235	0.025
			3	28-29w	0.000					
PNA spline * Sex	7.74	0.0005	1		0.000					
			2		-0.323	0.348	-1.006	0.361	-0.927	0.35
			3		0.270	0.071	0.131	0.409	3.801	0.0001

Suppl. Table 2.1. Statistical model for FiO_2 over postnatal age (PNA) using random coefficient models, with lognormal distribution and unstructured covariance pattern, including significant interactions. Time modeled with natural cubic splines.

Variable	F value	p-value	PNA spline	GA cat.	Estimate	SE	95% LCL	95% UCL	t-value	p-value
PNA spline	33.05	<.0001	1		0.000					
			2		-0.037	0.011	-0.059	-0.015	-3.277	0.0011
			3		0.006	0.004	-0.001	0.013	1.714	0.09
GA cat.	0.27	0.76		22-24w	0.032	0.047	-0.061	0.125	0.670	0.50
				25-27w	0.029	0.043	-0.055	0.113	0.677	0.50
				28-29w	0.000					
Sex	3.47	0.06			-0.039	0.021	-0.081	0.002	-1.863	0.06
PNA spline * GA cat.	8.13	<.0001	1	22-24w	0.000					
			1	25-27w	0.000					
			1	28-29w	0.000					
			2	22-24w	0.023	0.013	-0.004	0.049	1.694	0.09
			2	25-27w	0.007	0.013	-0.018	0.031	0.524	0.60
			2	28-29w	0.000					
			3	22-24w	0.011	0.004	0.003	0.019	2.635	0.0084
			3	25-27w	0.005	0.004	-0.002	0.013	1.385	0.17
			3	28-29w	0.000					

Suppl. table 2.2. Statistical model for alveolar-arterial (A-a) gradient over postnatal age (PNA) using random coefficient models, with lognormal distribution and unstructured covariance pattern, including significant interactions. Time modeled with natural cubic splines.

Variable	F value	p-value	PNA spline	GA cat.	Estimate	SE	95% LCL	95% UCL	t-value	p-value
PNA spline	21.14	<.0001	1		0.000					
			2		-0.073	0.028	-0.127	-0.018	-2.628	0.0087
			3		0.007	0.009	-0.010	0.023	0.773	0.44
GA cat.	4.53	0.011		22-24w	0.738	0.277	0.194	1.282	2.666	0.0079
				25-27w	0.713	0.251	0.219	1.206	2.838	0.0047
				28-29w	0.000					
Sex	0.38	0.54			0.164	0.129	-0.089	0.417	1.275	0.20
PNA spline * GA cat.	7.76	<.0001	1	22-24w	0.000					
			1	25-27w	0.000					
			1	28-29w	0.000					
			2	22-24w	0.037	0.034	-0.029	0.103	1.108	0.27
			2	25-27w	0.005	0.031	-0.056	0.066	0.158	0.87
			2	28-29w	0.000					
			3	22-24w	0.029	0.009	0.011	0.048	3.123	0.0018
			3	25-27w	0.014	0.009	-0.004	0.032	1.540	0.12
			3	28-29w	0.000					
GA cat * Sex	2.36	0.10		22-24w	-0.285	0.169	-0.618	0.048	-1.682	0.09
				25-27w	-0.322	0.150	-0.617	-0.026	-2.140	0.033
				28-29w	0.000					

Suppl. Table 3 (3.1 and 3.2). Relative risk (RR) with 95% CI from the statistical model for FiO_2 (A) and A-a gradient (B) as a function of HbF(%), pCO_2, gestational age (GA) and postnatal age (PNA), adjusted for pH and sex using random coefficient models, with lognormal distribution and unstructured covariance pattern, including significant interactions.

3.1: Estimates per 20 units increase in HbF(%)	RR (95% CI)	p-value
PNA 1d pCO2kPa(Q1)	0.99 (0.98 - 1.00)	0.19
PNA 1d pCO2kPa(Q3)	1.01 (0.99 - 1.02)	0.25
PNA 4d pCO2kPa(Q1)	0.94 (0.93 - 0.95)	<.0001
PNA 4d pCO2kPa(Q3)	0.96 (0.95 - 0.97)	<.0001
PNA 7d pCO2kPa(Q1)	0.89 (0.88 - 0.91)	<.0001
PNA 7d pCO2kPa(Q3)	0.91 (0.89 - 0.93)	<.0001

3.2: Estimates per 20 units increase in HbF(%)	RR (95% CI)	p-value
PNA 1d pCO2kPa(Q1)	1.01 (0.98 - 1.05)	0.42
PNA 1d pCO2kPa(Q3)	1.05 (1.01 - 1.08)	0.0065
PNA 4d pCO2kPa(Q1)	0.92 (0.89 - 0.94)	<.0001
PNA 4d pCO2kPa(Q3)	0.95 (0.92 - 0.97)	0.0002
PNA 7d pCO2kPa(Q1)	0.83 (0.79 - 0.87)	<.0001
PNA 7d pCO2kPa(Q3)	0.85 (0.82 - 0.89)	<.0001

Suppl. Table 4. Mean difference with 95% CI from the statistical model for arterial oxygen saturation (SaO_2) as a function of HbF(%), pCO_2, gestational age (GA) and postnatal age (PNA), adjusted for pH and sex using random coefficient models, with normal distribution and unstructured covariance pattern, including significant interactions. Q1 and Q3 of pCO_2 represent the 25th and 75th percentile respectively.

Estimates per 20 units increase in HbF(%)	LS mean difference (95% CI)	p-value
PNA 1d pCO2kPa(Q1)	-0.08 (-0.30 - 0.13)	0.45
PNA 1d pCO2kPa(Q3)	-0.16 (-0.38 - 0.05)	0.14
PNA 4d pCO2kPa(Q1)	0.14 (-0.04 - 0.31)	0.12
PNA 4d pCO2kPa(Q3)	0.06 (-0.11 - 0.23)	0.48
PNA 7d pCO2kPa(Q1)	0.36 (0.11 - 0.61)	0.0055
PNA 7d pCO2kPa(Q3)	0.28 (0.04 - 0.52)	0.022
